# Mixed-Phase Ion-Exchangers from Waste Amber Container Glass

**DOI:** 10.3390/ma14174887

**Published:** 2021-08-27

**Authors:** Victoria K. Elmes, Andrew P. Hurt, Nichola J. Coleman

**Affiliations:** School of Science, Faculty of Engineering and Science, University of Greenwich, Chatham Maritime, Kent ME4 4TB, UK; v.elmes@gre.ac.uk (V.K.E.); a.hurt@gre.ac.uk (A.P.H.)

**Keywords:** container glass, zeolites, sodalite, cancrinite, tobermorite, ion-exchange, recycling, hydrothermal synthesis

## Abstract

This study investigated the one-pot hydrothermal synthesis of mixed-phase ion-exchangers from waste amber container glass and three different aluminium sources (Si/Al = 2) in 4.5 M NaOH_(aq)_ at 100 °C. Reaction products were characterised by X-ray diffraction analysis, Fourier transform infrared spectroscopy, ^27^Al and ^29^Si magic angle spinning nuclear magnetic resonance spectroscopy and scanning electron microscopy at 24, 48 and 150 h. Nitrated forms of cancrinite and sodalite were the predominant products obtained with reagent grade aluminium nitrate (Al(NO_3_)_3_∙9H_2_O). Waste aluminium foil gave rise to sodalite, tobermorite and zeolite Na-P1 as major phases; and the principal products arising from amorphous aluminium hydroxide waste were sodalite, tobermorite and zeolite A. Minor proportions of the hydrogarnet, katoite, and calcite were also present in each sample. In each case, crystallisation was incomplete and products of 52, 65 and 49% crystallinity were obtained at 150 h for the samples prepared with aluminium nitrate (AN-150), aluminium foil (AF-150) and amorphous aluminium hydroxide waste (AH-150), respectively. Batch Pb^2+^-uptake (~100 mg g^−1^) was similar for all 150-h samples irrespective of the nature of the aluminium reagent and composition of the product. Batch Cd^2+^-uptakes of AF-150 (54 mg g^−1^) and AH-150 (48 mg g^−1^) were greater than that of AN-150 (36 mg g^−1^) indicating that the sodalite- and tobermorite-rich products exhibited a superior affinity for Cd^2+^ ions. The observed Pb^2+^- and Cd^2+^-uptake capacities of the mixed-product ion-exchangers compared favourably with those of other inorganic waste-derived sorbents reported in the literature.

## 1. Introduction

It is estimated that approximately 200 Mt of waste soda-lime-silica container glass are landfilled per annum [1]. In order to conserve energy and natural resources, it is theoretically possible to recycle up to 90% of waste container glass, although this potential is undermined by a range of geographical, economic and technical challenges [2,3,4]. In particular, poor collection infrastructure and colour mismatch restrict regional demand for coloured waste container glass that can be recycled as new bottles and jars. Accordingly, container glass recycling rates vary widely across the globe, with 42, 34 and 20% reported for Australia, USA and Singapore, respectively, and between 50 and 80% among the European countries [5]. To address the problems of landfilling and stockpiling post-consumer container glass, a number of recent studies has been carried out to reprocess this waste into value-added products, such as ceramics, ion-exchangers, catalysts, sorbents, geopolymers, alkali-activated cements and building materials [1,3,4,5,6,7,8,9,10,11,12,13,14,15].

Irrespective of colour, the principal oxide components of soda-lime-silica container glasses are SiO_2_ (66–75 wt%), Na_2_O (12–16 wt%), CaO (6–12 wt%), Al_2_O_3_ (0.7–7 wt%), MgO (0.1–5 wt%) and K_2_O (0.1–3 wt%), with trace chromophores (Fe_2_O_3_, SO_3_ and Cr_2_O_3_) below 0.5 wt% [16]. Hence, in comparison with other silicate wastes, such as slags and fly ashes, container glass of any origin provides a relatively predictable source of silica with negligible concentrations of toxic components [7,8,9]. The reactivity of the amorphous silica species in container glass under mild hydrothermal conditions has been exploited in several studies to produce a range of technologically relevant mineral phases including tobermorite (Ca_5_Si_6_O_16_(OH)_2_∙4H_2_O) [3,17,18,19], lithium metasilicate (Li_2_SiO_3_) [9,15,20] and various zeolites [6,7,8,9,14,21,22,23,24].

Zeolites and feldspathoids are 3-D microporous aluminosilicate framework materials of general formula M_x/n_[(AlO_2_)_x_(SiO_2_)_y_]_m_H_2_O (where n is the valence of the non-framework charge-balancing cation, and x, y and m are the relative moles of aluminium, silicon and water) [25]. Naturally occurring and synthetic zeolites find wide application in cosmetics and pharmaceuticals, catalysis, ion-exchange processes, adsorption and separation technologies, pollution control, soil conditioning, and animal feed [6,7,8,9,24,25,26]. The principal industrial roles of zeolites are largely based upon their ion-exchange and separation properties. The global market for zeolites is anticipated to reach 5.9 billion US dollars by 2023, and the current market for zeolites in the detergent industry is 1.4 billion US dollars alone [25]. Zeolites are included in laundry detergent formulations to exchange divalent cations for sodium ions to prevent the precipitation of surfactant salts (i.e., “scum”) [25]. Zeolites are also widely used in myriad industrial processes as desiccants for gases and liquids, particularly for the dehydration of solvents and fuels [25].

To date, impure low-silica zeolites (i.e., Si/Al molar ratio <2), such as A, F, P, X, sodalite, cancrinite and analcite, have been prepared from stoichiometrically adjusted mixtures of container glass and aluminium-bearing reagents in aqueous alkaline media under convection and microwave heating [6,7,8,9,14,21,22,23]. Typical one-step hydrothermal syntheses involve autoclaving ground glass (<2 mm) with an aluminium reagent (Si/Al molar ratio 1–10) in alkali metal hydroxide solution (0.5–8 M) between 60 and 200 °C for up to 14 days [6,7,8,9,14,21,22,23,24].

The present study extends the current research on the hydrothermal synthesis of container glass-based zeolites by considering the phase evolution of the reactions of amber container glass with three different aluminium-bearing sources (at Si/Al = 2) in 4.5 M NaOH_(aq)_ at 100 °C. The effect of using reagent grade aluminium nitrate (Al(NO_3_)_3_.9H_2_O) on the rate of crystallisation and product phase assembly was compared with those of waste aluminium foil (>99.9 wt% Al metal) and an amorphous aluminium hydroxide waste (~22.3 wt% Al) arising from the manufacture of extruded aluminium profiles. The crystallinity and composition of the reaction products were monitored at 24, 48 and 150 h by powder X-ray diffraction analysis (XRD) with Rietveld refinement, Fourier transform infrared spectroscopy (FTIR), ^29^Si and ^27^Al magic angle spinning nuclear magnetic resonance spectroscopy (MAS NMR) and scanning electron microscopy (SEM). The uptake of Pb^2+^ and Cd^2+^ ions by the 150-h reaction products were evaluated by batch sorption and compared with those of other low-cost and waste-derived inorganic sorbents.

## 2. Materials and Methods

### 2.1. Materials

Post-consumer amber soda-lime-silica beer bottles and aluminium foil were obtained from the municipal refuse in Kent, UK. The bottles and foil were rinsed with warm tap water. The foil was cut into 1 × 1 cm^2^ squares and the bottles were ground in a ball mill to pass 125 μm. Solid amorphous aluminium hydroxide waste arising from the manufacture of extruded aluminium profiles was obtained from Exlabesa, Campaña, Spain, and lightly ground by pestle and mortar to pass 250 μm. Quantitative compositional analyses of the amber container glass, aluminium foil and aluminium hydroxide waste were obtained by X-ray fluorescence spectroscopy (Materials Research Institute, Sheffield Hallam University, Sheffield, UK) and are listed in Table 1. The amber container glass and aluminium hydroxide waste were characterised by powder XRD, FTIR, ^27^Al MAS NMR, and the container glass was also characterised by ^29^Si MAS NMR (using the instruments and operating parameters described in Section 2.3). The characterisation data for the amber container glass are published elsewhere [14] and an X-ray diffraction pattern, FTIR spectrum, and ^27^Al MAS NMR spectrum of the amorphous aluminium hydroxide waste are located in Appendix A. All other reagents were obtained from Sigma-Aldrich, Gillingham, UK, and were used without further purification.

### 2.2. Hydrothermal Synthesis and Characterisation of Zeolites

The zeolite samples were prepared from 3.0 g of ground amber container glass combined with either 6.51 g of reagent grade aluminium nitrate nonahydrate (Al(NO_3_)_3_∙9H_2_O), 0.468 g of aluminium foil or 2.09 g of amorphous aluminium hydroxide waste to adjust the reaction mixture to Si/Al~2. Hydrothermal syntheses were carried out in triplicate by sealing the solid reagents and 15 cm^3^ of 4.5 M NaOH_(aq)_ in PTFE-lined autoclaves and heating at 100 °C. Samples prepared for 24, 48 and 150 h with aluminium nitrate were labelled AN-24, AN-48 and AN-150, respectively; and a similar labelling system was used for samples prepared from aluminium foil (*viz*. AF-24, AF-48, AF-150) and amorphous aluminium hydroxide waste (*viz*. AH-24, AH-48, AH-150). Reaction products were recovered by gravitational filtration, washed with deionised water to pH ~8 and dried to constant mass at 60 °C in air. The ground amber container glass, amorphous aluminium hydroxide waste and reaction products were analysed by powder XRD, FTIR, MAS NMR and SEM as described in [14,24].

### 2.3. Uptake of Pb^2+^ and Cd^2+^ Ions by the Zeolite Products

The kinetics of removal of divalent lead and cadmium ions from aqueous solutions by the hydrothermal reaction products, AN-150, AF-150 and AH-150, were evaluated in triplicate by single metal batch sorption experiments. In each case, 0.2 g of solid sample was added to 200 cm^3^ solution of either 0.5 mM Cd(NO_3_)_2_∙4H_2_O or 0.5 mM Pb(NO_3_)_2_ at 25 °C. 1 cm^3^ aliquots of the supernatant solutions were withdrawn at 0.5, 1, 3, 6 and 24 h and analysed by inductively coupled plasma spectroscopy (ICP) using a TJA Iris simultaneous ICP-OES spectrophotometer (TJA, New Bedford, MA, USA).

## 3. Results

### 3.1. Characterisation of the Zeolite Products

**Figure 1:**
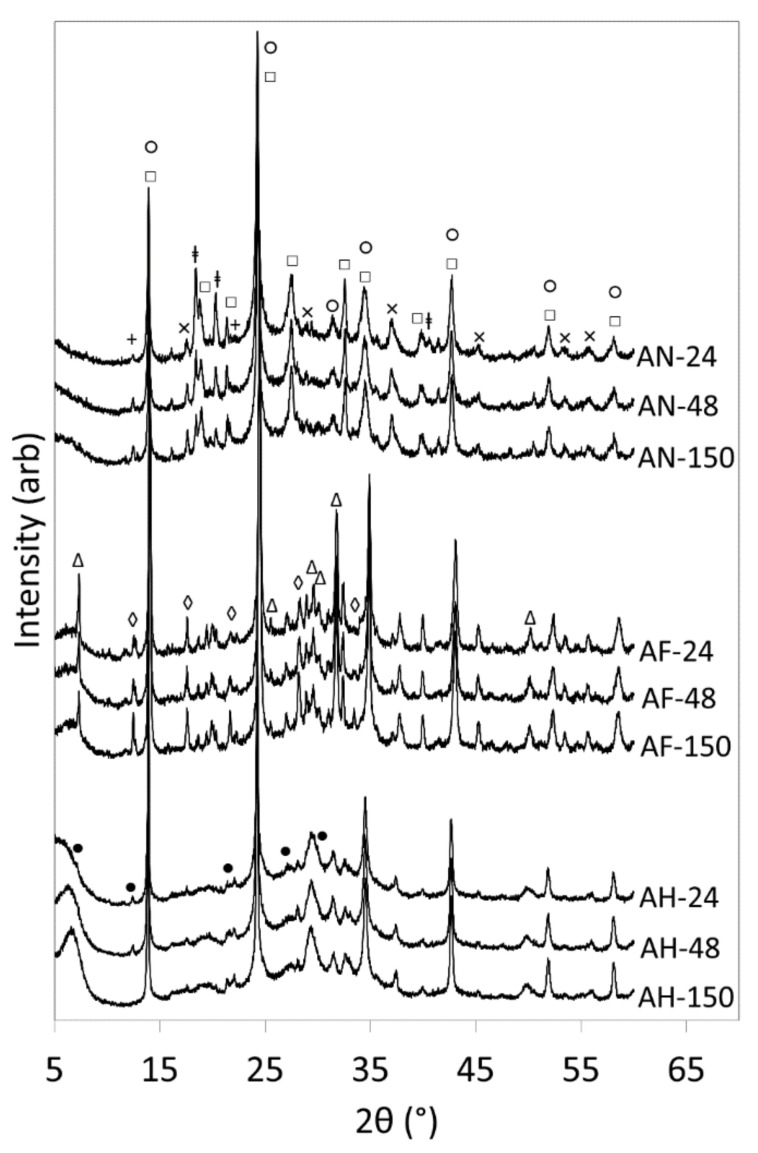
XRD patterns of hydrothermal products of amber container glass and aluminium nitrate (AN), aluminium foil (AF) and amorphous aluminium hydroxide waste (AH) synthesised for 24, 48 and 150 h in 4.5 M NaOH_(aq)_ at 100 °C. Key: ◌ sodalite; □ cancrinite; ∆ tobermorite; ◊ zeolite Na-P1; + zeolite P2; ● zeolite A; × katoite; ⱡ aluminium hydroxide.

X-ray diffraction patterns of the hydrothermal reaction products of waste amber container glass and aluminium nitrate are presented in Figure 1 and the corresponding sample compositions are listed in Table 2. These data indicate that, approximately 46% of the amorphous glass was transformed into crystalline reaction products within 24 h and that only a modest increase in crystallinity to 52% was achieved during the following 4 days (Table 2). Under the selected reaction conditions, the principal product phases were nitrate-enclathrated cancrinite and sodalite, at a constant mass ratio of ~1.2 irrespective of reaction time. Cancrinite and sodalite are ultramicroporous low-silica zeolites with different structural frameworks who share the common formula, Na(Al_6_Si_6_O_24_)∙2NaX.6H_2_O, where X is a mono- or divalent anion [24]. Minor proportions of zeolite P2 (ideal formula Na(Al_4_Si_12_O_32_)∙14H_2_O) and the hydrogarnet, katoite (Ca_3_Al_2_(SiO_4_)(OH)_8_), were also formed in this system. Aluminium hydroxide was initially precipitated from the aluminium nitrate reagent in the alkaline reaction liquor and incompletely consumed during hydrothermal processing; and atmospheric carbonation gave rise to trace quantities of calcite (Table 2).

Sodalite and the layer lattice ion-exchanger, tobermorite, were the predominant reaction products when aluminium foil (Figure 1, Table 3) or amorphous aluminium hydroxide waste (Figure 1, Table 4) were reacted with the amber container glass. In the latter case, minor proportions of zeolite A (Na_12_(Al_12_Si_12_O_24_)∙27H_2_O), katoite and calcite were also formed, and the rate of crystallisation was similar to that of the samples prepared with reagent grade aluminium nitrate. In addition to sodalite and tobermorite, the reaction of amber glass and aluminium foil gave rise to zeolite Na-P1 (Na_6_(Al_6_Si_10_O_32_)∙12H_2_O), katoite and calcite with the highest observed rate of crystallisation (Figure 1, Table 3).

**Figure 2:**
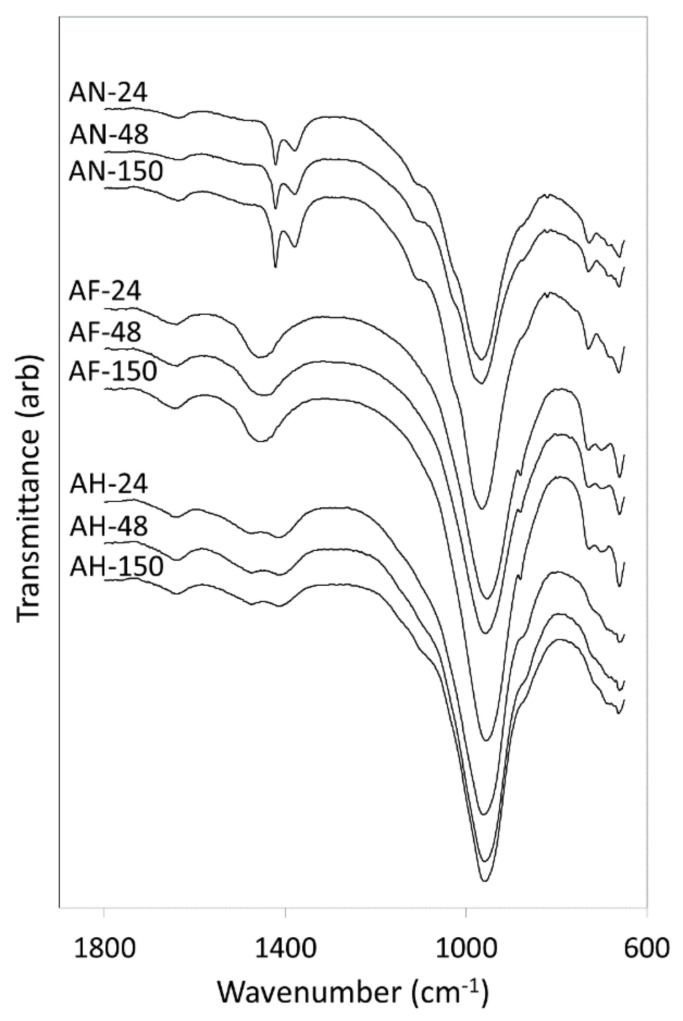
FTIR spectra of hydrothermal products of amber container glass and aluminium nitrate (AN), aluminium foil (AF) and amorphous aluminium hydroxide waste (AH) synthesised for 24, 48 and 150 h in 4.5 M NaOH_(aq)_ at 100 °C.

FTIR spectra of the hydrothermal reaction products of amber container glass and the three aluminium-bearing reagents are shown in Figure 2. In all cases, the broad band at 965 cm^−1^ arises from the antisymmetric Si(Al)-O-Si stretching vibrations of the aluminosilicate frameworks of the zeolite products and the amorphous silicate network of the residual unreacted amber glass [14]. Symmetric stretching of framework Si(Al)-O-Si appears at 730 cm^−1^ and O-Si(Al)-O bending vibrations occur at 690 and 660 cm^−1^ [24]. The broad signal at 1640 cm^−1^ is attributed to the bending modes of water and hydroxyl ions, and carbonate ion stretching gives rise to the signals *circa* 1450 cm^−1^. Evidence for the enclathration of the nitrate anion in the sodalite and cancrinite products formed during the reaction of aluminium nitrate and amber glass is provided by the asymmetric stretching modes of NO_3_^−^ at 1378 and 1422 cm^−1^ in the spectra labelled AN-24, AN-48 and AN-150 (Figure 2) [27].

**Figure 3:**
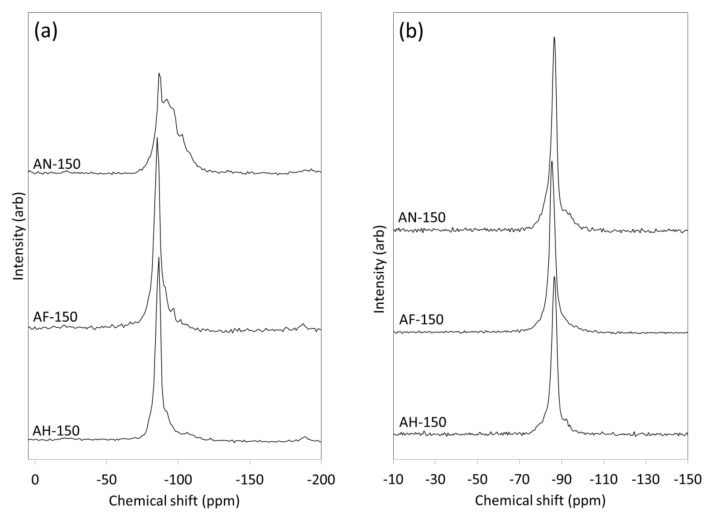
(**a**) ^29^Si MAS NMR spectra and (**b**) ^1^H-^29^Si CP MAS NMR spectra of hydrothermal products of amber container glass and aluminium nitrate (AN-150), aluminium foil (AF-150) and amorphous aluminium hydroxide waste (AH-150) synthesised for 150 h in 4.5 M NaOH_(aq)_ at 100 °C.

The single pulse ^29^Si MAS NMR spectra and ^1^H-^29^Si cross-polarization (CP) MAS NMR spectra of the reaction products of amber container glass and the three aluminium-bearing reagents after 150 h are shown in Figure 3. The single pulse ^29^Si MAS NMR spectrum of the reaction products of amber glass and aluminium nitrate (AN-150) comprises a sharp resonance at −86.5 ppm superposed over a broader signal of maximum intensity ~−91 ppm (Figure 3a). The former resonance is characteristic of the framework silicate units in nitrate-enclathrated cancrinite and sodalite [28] and the latter broader downfield signal is attributed to residual unreacted glass and also to the formation of a calcium/sodium aluminosilicate gel phase [24]. The corresponding ^1^H-^29^Si CP MAS NMR spectrum (AN-150, Figure 3b) shows only the hydrated product phases in which the broad underlying signal between −75 and −98 ppm confirms the presence of an amorphous aluminosilicate gel phase.

The asymmetrical resonance at −85.5 ppm in the single pulse ^29^Si MAS NMR spectrum of the reaction products of amber glass and aluminium foil (AF-150, Figure 3a) arises from the framework Si(OAl)_4_ tetrahedra in sodalite and zeolite Na-P1, and the various unresolved silicate species within the wollastonite-like chains of the tobermorite phase [3]. Residual parent glass is evident as a downfield shoulder on the central resonance that is absent from the corresponding ^1^H-^29^Si CP MAS NMR spectrum (AF-150, Figure 3b). The proportions of residual glass and amorphous aluminosilicate gel present in the spectra of sample AF-150 are considerably lower than those of sample AN-150 and correspond well with the XRD data that confirmed the superior crystallinity of the sample derived from aluminium foil (Figure 1, Table 2 and Table 3).

The single pulse ^29^Si MAS NMR spectrum of the reaction products of amber glass and amorphous aluminium hydroxide waste (AH-150, Figure 3a) presents an asymmetrical signal of maximum intensity at −88.7 ppm which is assigned to unresolved contributions from the silicate species in sodalite, tobermorite and zeolite A [3,29]. Residual glass and an aluminosilicate gel phase appear as a broad underlying signal (AH-150, Figure 3a), and the aluminosilicate gel phase is also visible in the corresponding ^1^H-^29^Si CP MAS NMR spectrum (AH-150, Figure 3b). 

**Figure 4:**
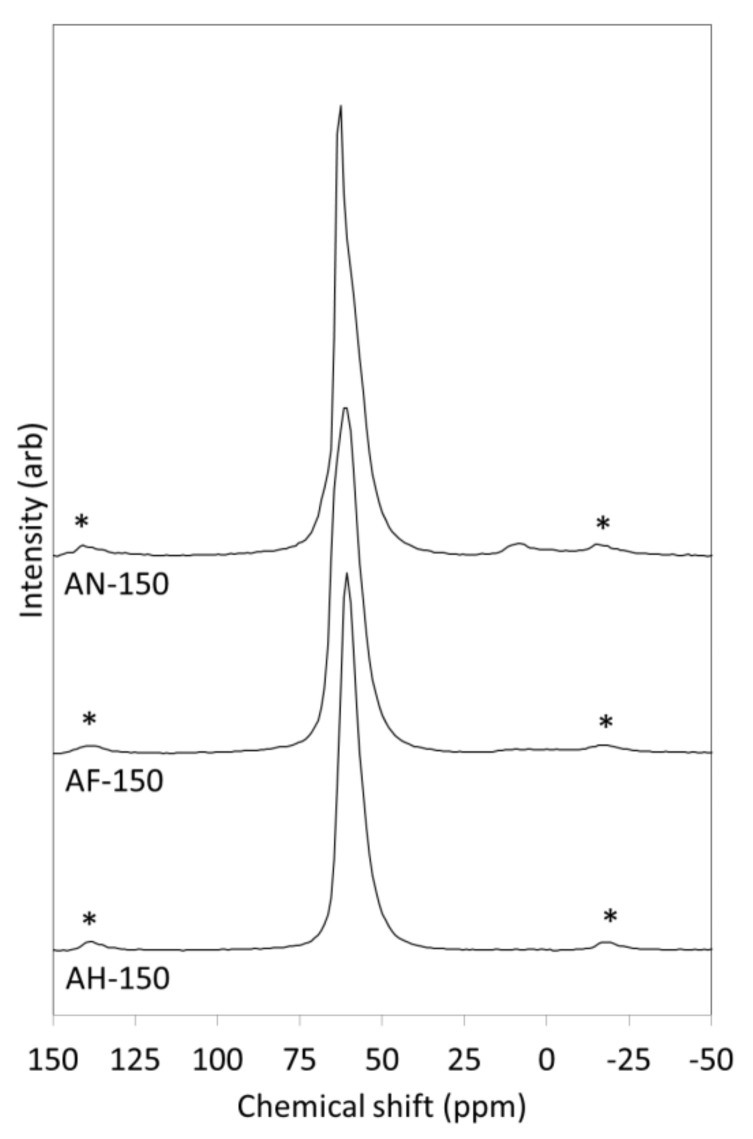
^27^Al MAS NMR spectra of hydrothermal products of amber container glass and aluminium nitrate (AN-150), aluminium foil (AF-150) and amorphous aluminium hydroxide waste (AH-150) synthesised for 150 h in 4.5 M NaOH_(aq)_ at 100 °C. (Spinning side bands are denoted by asterisks.)

The asymmetrical signal in the ^27^Al NMR spectrum of AN-150 at ~62 ppm (Figure 4) arises from the tetrahedral aluminate species in the nitrate-enclathrated cancrinite and sodalite with contributions from zeolite P2 and residual amber glass [24]. The weaker very broad octahedral signal circa 12 ppm is assigned to the octahedral aluminium in katoite [24]. The ^27^Al NMR spectra of AF-150 and AH-150 (Figure 4) are characterised by broad tetrahedral resonances at ~60 ppm arising from the various unresolved aluminium environments in sodalite and tobermorite and also from the minor zeolite products [24].

**Figure 5:**
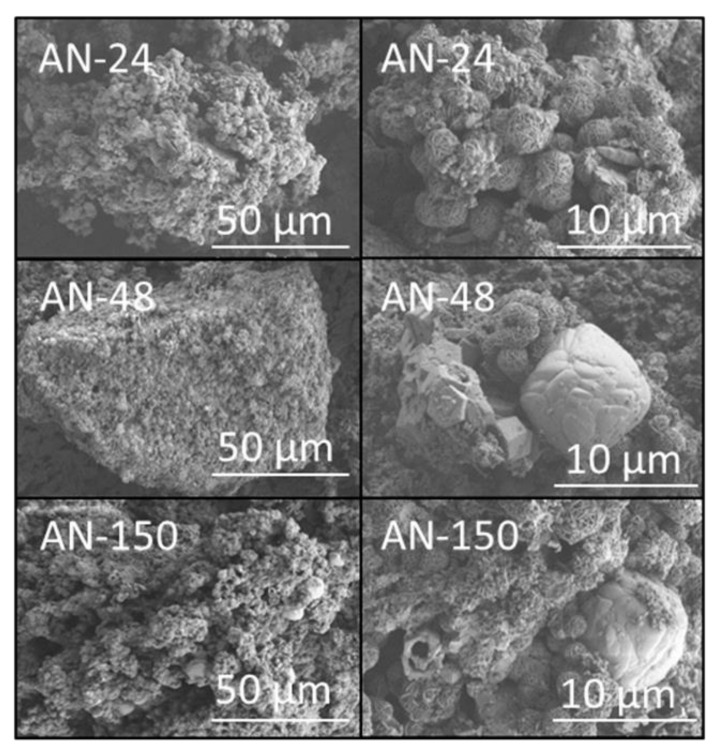
Secondary electron SEM images of hydrothermal products of amber container glass and aluminium nitrate (AN) synthesised for 24, 48 and 150 h in 4.5 M NaOH_(aq)_ at 100 °C.

**Figure 6:**
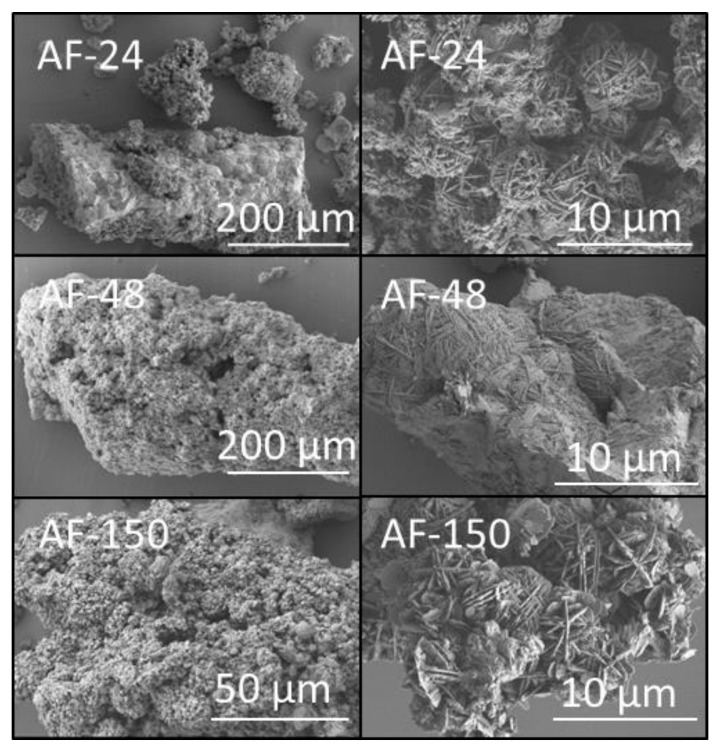
Secondary electron SEM images of hydrothermal products of amber container glass and aluminium foil (AF) synthesised for 24, 48 and 150 h in 4.5 M NaOH_(aq)_ at 100 °C.

**Figure 7:**
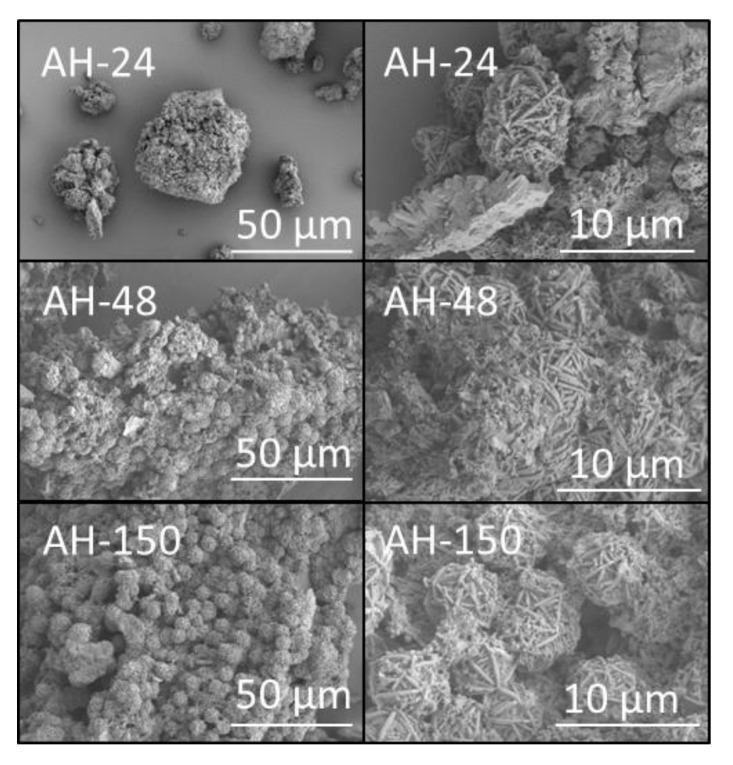
Secondary electron SEM images of hydrothermal products of amber container glass and amorphous aluminium hydroxide waste (AH) synthesised for 24, 48 and 150 h in 4.5 M NaOH_(aq)_ at 100 °C.

Secondary electron SEM images of the hydrothermal reaction products of amber glass and aluminium nitrate, aluminium foil or amorphous aluminium hydroxide waste are presented in Figure 5, Figure 6 and Figure 7, respectively. In all cases, the hydrothermal processing of amber glass with the various aluminium-bearing reagents resulted in granular products of broad particle size distribution up to approximately 500 μm (Figure 5, Figure 6 and Figure 7). The surfaces of the materials derived from aluminium nitrate were largely populated with characteristic ball-of-wool sodalite clusters and hexagonal nut-like cancrinite precipitates between 1 and 5 μm in diameter (Figure 5). Larger globular deposits (~10 μm) of sodium/calcium aluminosilicate gel were also dispersed across the surfaces of samples AN-48 and AN-150 (Figure 5) [14].

The surfaces of the products derived from aluminium foil (Figure 6) and amorphous aluminium hydroxide waste (Figure 7) were extensively covered with interpenetrating clusters of sodalite up to 10 μm in diameter; and occasional discrete foils of tobermorite were also observed on the surface of sample AF-150 (Figure 6).

### 3.2. Ion-Exchange Properties of the Zeolite Products

**Figure 8:**
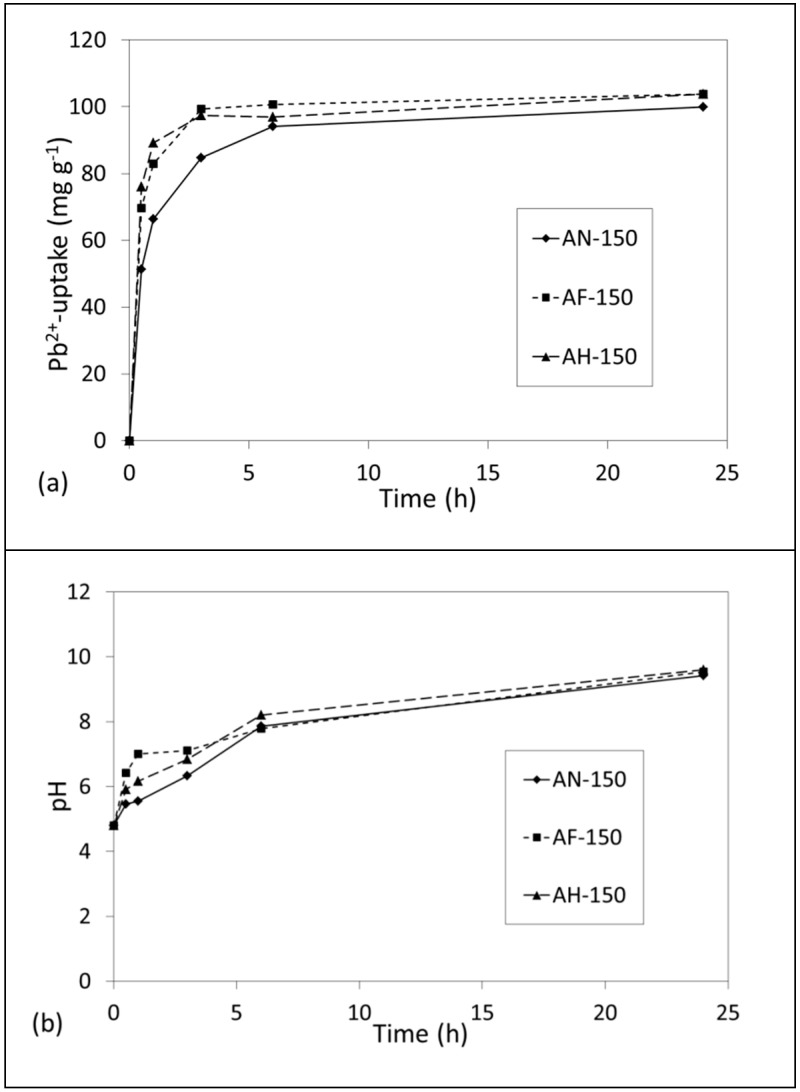
(**a**) Removal of Pb^2+^ ions by hydrothermal products AN-150, AF-150 and AH-150 and (**b**) corresponding pH values of the supernatant liquors.

**Figure 9:**
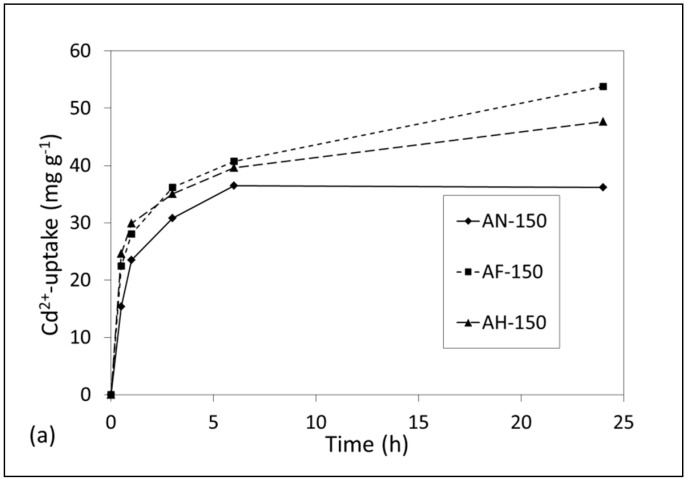

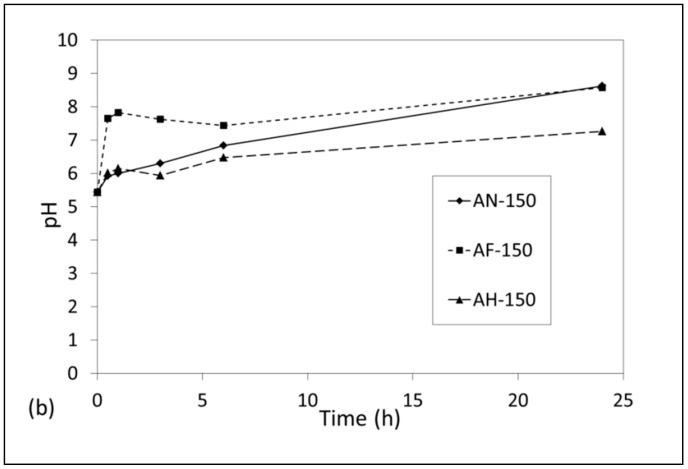
(**a**) Removal of Cd^2+^ ions by hydrothermal products AN-150, AF-150 and AH-150 and (**b**) corresponding pH values of the supernatant liquors.

The uptake of Pb^2+^ and Cd^2+^ ions from single metal ion solutions by AN-150, AF-150 and AH-150 and the corresponding pH values of the supernatant liquors are plotted in Figure 8 and Figure 9. Equilibrium uptake of Pb^2+^ ions by all samples (~100 mg g^−1^, ~0.48 mmol g^−1^) was established within 6 h at an efficiency of greater than 99% (Figure 8a). Marked increases in pH from an initial value of 4.8 accompanied the removal of Pb^2+^ ions as charge-balancing Ca^2+^ and Na^+^ ions were exchanged into the solution (Figure 8b). Supernatant pH continued to increase at a slower rate beyond the point of equilibrium uptake to give a final solution pH of 9.6 at 24 h.

Equilibrium removal of Cd^2+^ ions by AN-150 (36 mg g^−1^, 0.32 mmol g^−1^) was observed within 6 h at an efficiency of 67% (Figure 9a). Conversely, maximum Cd^2+^-uptakes of AF-150 (54 mg g^−1^, 0.48 mmol g^−1^) and AH-150 (48 mg g^−1^, 0.43 mmol g^−1^) were greater and the equilibrium times were longer (Figure 9a). Previous research has demonstrated that the uptake of Cd^2+^ ions by tobermorite is high, ~180 mg g^−1^, but relatively slow, with equilibrium times of several days [30] which accounts for the difference in uptake profiles between sample AN-150 and the tobermorite-bearing AF-150 and AH-150 products. Increases in supernatant Cd^2+^ solution pH from an initial value of 5.4 to 7.3 for AH-150, and to 8.6 for both AN-150 and AF-150 were noted, although the trends did not correlate directly with the observed extents of Cd^2+^-removal.

The batch uptakes of Pb^2+^ and Cd^2+^ by AN-150, AF-150 and AH-150 are compared with those of other low-cost and waste-derived inorganic sorbents in Table 5 [3,30,31,32,33,34,35,36,37,38,39,40]. The Pb^2+^ removal capacities and equilibrium times of the amber glass-derived mixed-phase sorbents were superior to those reported for waste concretes [31,32] and inferior to those of fly ash-derived zeolite Na-X [33] and hydrated calcium silicate gel [35]. Maximum Pb^2+^ uptake of glass-derived tobermorite [3] was approximately 3.5 times higher than that of the glass-derived mixed-phase sorbents, although the reported tobermorite sample failed to achieve equilibrium within 24 h. Similarly, the Cd^2+^ removal capacity of waste-derived tobermorite [30] was greater than that of AN-150, AF-150 and AH-150 with a significantly longer equilibrium time of greater than 6 days.

In general, the batch uptakes of Pb^2+^ and Cd^2+^ by AN-150, AF-150 and AH-150 and their associated equilibrium times fall within the ranges reported for other low-cost and waste-derived inorganic materials [3,30,31,32,33,34,35,36,37,38,39,40].

## 4. Discussion

Recent studies have indicated that soda-lime-silica container glass is a potentially useful feedstock for the facile one-step hydrothermal synthesis of various impure zeolites [6,7,8,9,14,21,22,23,24,25]. In the absence of pre-conditioning, the presence of 6–12 wt% CaO in container glass restricts the hydrothermal products to small-pore low-silica zeolites that tolerate in situ Ca^2+^ incorporation during crystallisation and also gives rise to other, more dense, calcium aluminosilicate phases such as tobermorite and katoite [9,14].

Irrespective of the reported particle size of the container glass (0.1–2 mm), hydroxide concentration (0.5–8 M), nature of the aluminium reagent, Si/Al ratio (1–10), and reaction temperature (60 and 200 °C), the rates of product crystallization are slow under conventional hydrothermal heating and rarely achieve more than 60% within 24 h [6,7,8,9,14,21,22,23,24]. On a laboratory scale, microwave heating has been used to markedly enhance the crystallization rate of zeolites derived from container glass [6,7,8]. For example, Manisab et al. [7] report that a 60% crystalline mixture of analcite, hydroxysodalite and zeolite NaP was produced under conventional heating at 150 °C for 24 h from 1.8 g of glass, 2.55 g of sodium aluminate and 36 cm^3^ of 0.5 M NaOH_(aq)_, and that a similar degree of crystallinity could be obtained from the same reagents within 10 min using microwave assisted synthesis. Despite the dramatic reduction in crystallization time that has been demonstrated on small samples in the laboratory, in practice, the scale-up of mineral synthesis using microwave heating is beset with problems [42,43]. In particular, low microwave penetration depths (i.e., a few centimetres at 2.45 GHz), inhomogeneous dissipation of energy, localized overheating, and poor reproducibility limit the scale-up of microwave assisted mineral synthesis [42,43]. In addition, the application of microwaves also prohibits the direct use of metallic reagents such as scrap aluminium (e.g., foil, cans, and profiles) without a pre-digestion step.

Long crystallization times associated with the hydrothermal synthesis of zeolites are reported to be reduced by a factor of up to five by the incorporation of oxyanionic promoter ions (e.g., PO_4_^3−^, AsO_4_^3−^, CO_3_^2−^, SO_4_^2−^, ClO_4_^−^, NO_3_^−^, ClO_3_^−^) [44]. This effect is attributed the ability of the oxyanions to promote condensation reactions and to stabilize the oligomeric silicate ions responsible for nucleation and growth [44]. To date, the use of promoter ions has not been investigated with respect to their potential to accelerate the crystallization kinetics of zeolites from container glass. Accordingly, the present study compared the phase evolution of hydrothermal products from mixtures of container glass with discarded aluminium foil, amorphous aluminium hydroxide waste or reagent grade aluminium nitrate (in order to incorporate promoter NO_3_^−^ ions without altering the Na:Ca:Si:Al reaction ratio).

The nature of the aluminium reagent was found to have a profound influence on the crystalline products under the selected reaction conditions (i.e., direct one-step hydrothermal processing, without pre-conditioning or pre-gelling, at Si/Al = 2 in 4.5 M NaOH_(aq)_ at 100 °C for 1, 2 and 5 days). Nitrated forms of cancrinite and sodalite were the predominant products obtained with reagent grade aluminium nitrate (Table 2). Waste aluminium foil gave rise to sodalite, tobermorite and zeolite Na-P1 as major phases (Table 3); and the principal products arising from amorphous aluminium hydroxide waste were sodalite, tobermorite and zeolite A (Table 4). As anticipated, minor proportions of the hydrogarnet, katoite, and calcite were also present in each sample.

Crystallization rates were observed to be of the following order AF > AH = AN, indicating that the incorporation of NO_3_^−^ promoter ions did not enhance the reaction kinetics (Table 2, Table 3 and Table 4). In fact, greater proportions of residual parent glass and aluminosilicate gel phase were associated with the aluminium nitrate reagent, indicating that the NO_3_^−^ ions may play a role in stabilizing the amorphous material in this system (Figure 3). 

An extensive study on the impact of Na^+^ and SO_4_^2−^ ions on the hydrothermal synthesis of zeolite A indicates that it is, in fact, the concentration of the ‘structure-forming’ sodium cation rather than the presence of the sulphate oxyanion that accelerates the kinetics of crystallization [45]. The present study is in tentative agreement with this viewpoint, as no advantage in crystallization kinetics was observed in the nitrate-bearing system. More typically, NO_3_^−^ ions are acknowledged to favour the crystallization of cancrinite over sodalite which accounts for its exclusive appearance among the major products in the system containing aluminium nitrate [27,45].

Since many technical and industrial applications of waste-derived low-silica zeolites depend upon their high ion-exchange capacities, aspects of the ion-exchange characteristics of the mixed-phase products were considered in this study. Batch Pb^2+^-uptake of ~100 mg g^−1^ was found to be similar for all 150-h samples irrespective of the nature of the aluminium reagent and composition of the product. Conversely, batch Cd^2+^-uptakes of AF-150 (54 mg g^−1^) and AH-150 (48 mg g^−1^) were observed to be greater than that of AN-150 (36 mg g^−1^) indicating that the sodalite- and tobermorite-rich products exhibited a superior affinity for Cd^2+^ ions. In general, the Pb^2+^- and Cd^2+^-uptake capacities of the mixed-product ion-exchangers were found to compare favourably with those of other inorganic waste-derived sorbents reported in the literature.

## 5. Conclusions

This study has demonstrated that mixed-phase cation-exchangers can be prepared from amber container glass and solid waste aluminium sources by a one-step hydrothermal reaction (Si/Al = 2 in 4.5 M NaOH_(aq)_ at 100 °C). Waste amorphous aluminium hydroxide from the manufacture of aluminium profiles gave rise sodalite, tobermorite and zeolite A; and the principal products arising from discarded aluminium foil were sodalite, tobermorite and zeolite Na-P1. Nitrated forms of cancrinite and sodalite were the predominant products obtained with reagent grade aluminium nitrate, and the presence of nitrate ‘promoter’ ions in this system did not accelerate the formation of the zeolites.

In all cases, crystallisation was incomplete and products of 52, 65 and 49% crystallinity were obtained at 150 h for the samples prepared with aluminium nitrate (AN-150), aluminium foil (AF-150) and amorphous aluminium hydroxide waste (AH-150), respectively. Batch Pb^2+^-uptake of ~100 mg g^−1^ was similar for all 150-h samples irrespective of the nature of the aluminium reagent and composition of the product. Conversely, batch Cd^2+^-uptakes of AF-150 (54 mg g^−1^) and AH-150 (48 mg g^−1^) were greater than that of AN-150 (36 mg g^−1^) indicating that the sodalite- and tobermorite-rich products, derived exclusively from waste materials, exhibited a superior affinity for Cd^2+^ ions.

## Figures and Tables

**Table 1:** Compositions of waste container glass, aluminium foil and amorphous aluminium hydroxide waste.

Element	Amber Container Glass (wt%)	Aluminium Foil (wt%)	Aluminium Hydroxide Waste (wt%)
Si	33.1	-	0.43
Al	1.17	>99.9	22.3
O	46.4	trace	59.8
Na	10.2	-	0.29
Ca	7.17	-	1.23
K	0.72	-	-
Mg	0.86	-	-
C	-	-	10.5
Fe	0.30	-	1.44
S	0.12	-	3.41
Cl	-	-	0.24
Cr	0.03	-	-
Sn	-	-	0.40

**Table 2:** Compositions of the hydrothermal reaction products of amber container glass and aluminium nitrate.

Phase	AN-24	AN-48	AN-150
Cancrinite nitrate (PDF 01-071-2841) (%)	42.2	45.4	48.7
Sodalite nitrate (PDF 00-050-0248) (%)	38.1	38.9	40.3
Zeolite P2 (PDF 01-080-0700) (%)	0.82	3.13	3.86
Katoite (PDF 01-076-2504) (%)	4.45	4.62	5.30
Aluminium hydroxide (01-0806432) (%)	13.8	7.41	1.75
Calcite (PDF 00-066-0867) (%)	0.62	0.51	-
Crystallinity (%)	45.8 ± 7.2	49.8 ± 0.3	51.9 ± 0.9
Weighted profile R-factor (R_wp_)	5.69	5.45	5.43

The proportion of each product phase is expressed as a percentage of the total mass of crystalline material within the sample.

**Table 3:** Compositions of the hydrothermal reaction products of amber container glass and aluminium foil.

Phase	AF-24	AF-48	AF-150
Sodalite (PDF 00-073-4004) (%)	65.6	58.7	63.4
Tobermorite (PDF 01-019-0052) (%)	14.2	18.8	13.3
Zeolite Na-P1 (PDF 01-071-0962) (%)	6.15	9.83	10.1
Katoite (PDF 01-076-2504) (%)	10.4	8.17	9.23
Calcite (PDF 00-066-0867) (%)	3.70	4.43	4.01
Crystallinity (%)	60.1 ± 0.2	61.8 ± 0.1	65.0 ± 0.1
Weighted profile R-factor (R_wp_)	14.5	14.1	14.9

The proportion of each product phase is expressed as a percentage of the total mass of crystalline material within the sample.

**Table 4:** Compositions of the hydrothermal reaction products of amber container glass and waste aluminium hydroxide.

Phase	AH-24	AH-48	AH-150
Sodalite (PDF 00-073-4004) (%)	68.5	72.6	70.8
Tobermorite (PDF 01-019-0052) (%)	22.5	17.9	19.8
Zeolite A (PDF 00-073-2340) (%)	4.06	5.22	4.63
Katoite (PDF 01-076-2504) (%)	1.05	0.52	0.90
Calcite (PDF 00-066-0867) (%)	3.87	3.76	3.87
Crystallinity (%)	43.5 ± 5.2	45.8 ± 0.6	48.7 ± 0.8
Weighted profile R-factor (R_wp_)	3.48	4.04	4.52

The proportion of each product phase is expressed as a percentage of the total mass of crystalline material within the sample.

**Table 5:** Comparison of the uptakes of Pb^2+^ and Cd^2+^ by AN-150, AF-150, AH-150 and those of other low-cost and waste-derived inorganic sorbents.

Sorbent	^1^*C_i_* Range (ppm)	Solid:Liquid Ratio (mg cm^−3^)	^2^*q_m_* (mg g^−1^)	^3^*t_eq_* (min)	Ref
**Lead, Pb^2+^**					
AN-150	104	1	100	360	This study
AF-150	104	1	104	360	This study
AH-150	104	1	104	360	This study
Crushed concrete fines	1000	25	37.9	2880	[31]
Thermally modified concrete	5–1500	2–50	73.83	1440	[32]
Fly ash-derived zeolite Na-X	10–200	0.3	575	180	[33]
Slag-derived geopolymer	5–500	12.5	83.2	-	[34]
Hydrated calcium silicate gel	50–300	20	263	180	[35]
Glass-derived tobermorite	104	0.25	344	>1440	[3]
Natural glauconite	5–220	12.5	9.12	180	[36]
**Cadmium, Cd^2+^**					
AN-150	56	1	36.5	360	This study
AF-150	56	1	53.8	>1440	This study
AH-150	56	1	47.7	>1440	This study
Blast furnace slag	0-5	0.1–20	5.1	1440	[37]
Natural zeolite	80–600	20–320	25.9	20	[38]
Natural glauconite	5–220	12.5	3.44	180	[36]
Crushed concrete fines	10–1500	25	45.2	7200	[39]
Waste-derived calcium silicate	200	2.5	70.8	180	[40]
Waste-derived tobermorite	5.6–124	0.25	179	8640	[30]
Waste-derived calcium silicate	100–10000	25	198	1	[41]

^1^ *C_i_* = initial metal concentration in solution. ^2^ *q_m_* = maximum metal uptake. ^3^ *t_eq_* = time to equilibrium.

**Figure A1:**
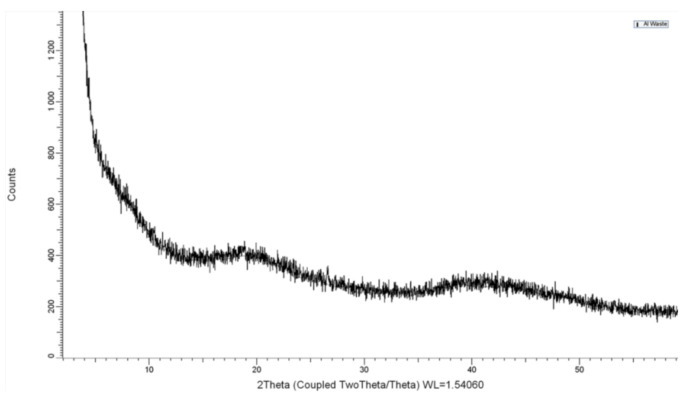
XRD pattern of the amorphous aluminium hydroxide waste.

**Figure A2:**
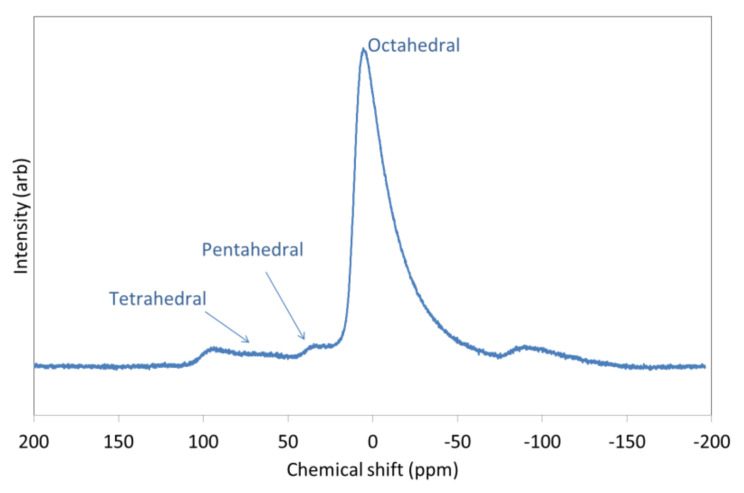
^27^Al MAS NMR spectrum of the amorphous aluminium hydroxide waste.

**Figure A3:**
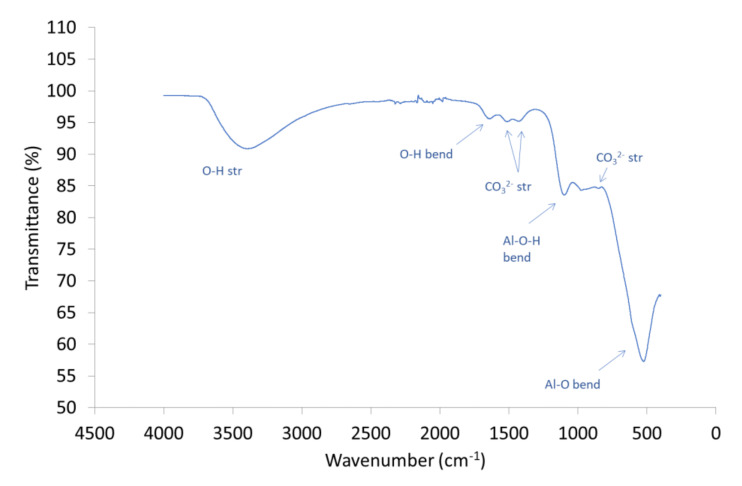
FTIR spectrum of the amorphous aluminium hydroxide waste.

## Data Availability

The data presented in this study are available on request from the corresponding author. The data are not publicly available due to intellectual property and privacy issues.

## Appendix A

### Characterisation of the Amorphous Aluminium Hydroxide Waste

An X-ray diffraction pattern, ^27^Al MAS NMR spectrum and FTIR spectrum of the amorphous aluminium hydroxide waste are given in Figure A1, Figure A2 and Figure A3. The XRD data confirm that this material is amorphous. The ^27^Al MAS NMR spectrum resembles that of gibbsite (Al(OH)_3_) that has been heated between 200 and 350 °C [46] and, in addition to aluminium hydroxide, the FTIR spectrum depicts the presence of carbonate ions.
